# Machine-learning-based coordination of powered ankle–foot orthosis and functional electrical stimulation for gait control

**DOI:** 10.3389/fbioe.2023.1272693

**Published:** 2024-01-10

**Authors:** Suhun Jung, Jae Hwan Bong, Keri Kim, Shinsuk Park

**Affiliations:** ^1^ Artificial Intelligence and Robot Institute, Korea Institute of Science and Technology, Seoul, Republic of Korea; ^2^ Department of Human Intelligence Robot Engineering, Sangmyung University, Cheonan-si, Republic of Korea; ^3^ Augmented Safety System With Intelligence, Korea Institute of Science and Technology, Seoul, Republic of Korea; ^4^ Division of Bio-Medical Science and Technology, University of Science and Technology, Daejeon, Republic of Korea; ^5^ Department of Mechanical Engineering, Korea University, Seoul, Republic of Korea

**Keywords:** powered ankle-foot orthosis (PAFO), functional electrical stimulation (FES), gait rehabilitation, machine learning, volitional electromyography (EMG)

## Abstract

This study proposes a novel gait rehabilitation method that uses a hybrid system comprising a powered ankle–foot orthosis (PAFO) and FES, and presents its coordination control. The developed system provides assistance to the ankle joint in accordance with the degree of volitional participation of patients with post-stroke hemiplegia. The PAFO adopts the desired joint angle and impedance profile obtained from biomechanical simulation. The FES patterns of the tibialis anterior and soleus muscles are derived from predetermined electromyogram patterns of healthy individuals during gait and personalized stimulation parameters. The CNN-based estimation model predicts the volitional joint torque from the electromyogram of the patient, which is used to coordinate the contributions of the PAFO and FES. The effectiveness of the developed hybrid system was tested on healthy individuals during treadmill walking with and without considering the volitional muscle activity of the individual. The results showed that consideration of the volitional muscle activity significantly lowers the energy consumption by the PAFO and FES while providing adaptively assisted ankle motion depending on the volitional muscle activities of the individual. The proposed system has potential use as an assist-as-needed rehabilitation system, where it can improve the outcome of gait rehabilitation by inducing active patient participation depending on the stage of rehabilitation.

## 1 Introduction

Stroke is a common geriatric disease caused by an interruption in the blood supply to the brain. It is usually accompanied by physical, neurological, and emotional dysfunctions, which greatly degrade the patient’s quality of life. The most common symptom of stroke is foot drop. It is a secondary hemiplegic and foot-dragging condition attributed to the abnormal dorsiflexion of the ankle. Rehabilitation of stroke patients should include treatment for foot drop to improve their gait ability in daily life (Fernandes et al., 2006; Kottink et al., 2010). Traditional treatments for foot drop are characterized by passive joint movements assisted by multiple therapists. Treatment efficiency is heavily dependent on the skill and experience of the therapist. As there is a critical time window for the rehabilitation of post-stroke patients, the shortage of experienced therapists with traditional rehabilitation treatments makes it difficult for them to receive proper and timely treatment (Leach et al., 2010; Song et al., 2016).

Functional electrical stimulation (FES) generates active joint movements by stimulating the nerves or muscles through a series of low-energy electrical impulses. FES has been used in the rehabilitation of the upper (Chadwick et al., 2011; Cooman and Kirsch, 2012) and lower limbs (Downey et al., 2013; Chen et al., 2014; Aksöz et al., 2016), where it can be easily applied to produce movement. However, its therapeutic effects remain controversial (Hardin et al., 2007). Liberson (1961) first applied FES to assist the gait of patients with hemiplegia. Subsequent studies investigated the possibility of producing natural gait patterns using FES (O'Keeffe et al., 2003; Sabut et al., 2010). O'Keeffe et al. demonstrated that the dorsiflexion angle could be increased with less energy consumption by applying FES patterns that resemble normal electromyography (EMG) patterns during gait (O'Keeffe et al., 2003). In their study, the FES patterns were simply mapped from the EMG profile during normal gait, without considering the underlying mechanism that relates FES to EMG.

Although a few attempts have been made to determine the direct relationship between FES and EMG, it can be modeled indirectly based on the results of other studies. Several studies have investigated the relationship between EMG and joint torque using musculoskeletal models and machine-learning techniques (Shin et al., 2009; Gui et al., 2019; Kim et al., 2020). Ito et al. (2007) proposed a linear model for FES and joint torques. In a previous study, we developed a model associating EMG and FES by combining the relationships between EMG and joint torque and between joint torque and FES (Jung et al., 2021).

Recently, machine-learning techniques have been used in various fields to make predictions and decisions based on training data. Among these, deep neural networks (DNNs) have demonstrated their effectiveness in handling highly nonlinear problems. Several studies have applied DNN to develop nonlinear models for estimating and predicting EMG signals (Hahn, 2007; Kordjazi and Rahati, 2012; Li et al., 2016). Among the various types of DNN structures, a one-dimensional convolutional neural network (1D CNN) is known to exhibit superior performance in capturing the features of dynamic time-series data, such as EMG (Ordóñez and Roggen, 2016; Yang et al., 2019; Jung et al., 2021).

Exoskeleton-type assistive devices have been widely used in gait rehabilitation of post-stroke patients (Gordon and Ferris, 2007; Shorter et al., 2011; Lv et al., 2016; Gil et al., 2018; Koller et al., 2018). A powered ankle–foot orthosis (PAFO) is a wearable exoskeleton that assists in the dorsiflexion and plantar flexion of the ankle joint (Gordon and Ferris, 2007; Lv et al., 2016; Koller et al., 2018). To ensure safety and backdrivability, many PAFO systems use impedance control to modulate the joint stiffness while tracking the desired joint motion (Emmens et al., 2018; Moltedo et al., 2018). Impedance control allows the actuator to react adaptively to the patient motion. The EMG signal of the patient can be used as an input to the impedance controller to detect the motion intention and state of the patient (Karavas et al., 2015; Huo et al., 2016; Alouane et al., 2019).

Recent studies have begun to investigate combined systems of FES and exoskeleton, which have both advantages and disadvantages as actuation systems. FES devices are lightweight and operate with a low power supply. However, the muscle force generated by FES is highly variable and difficult to control because it depends on the muscle condition. Although joint torque and motion can be precisely controlled with actuators equipped in an exoskeletal system, the heavy weight of the system makes it difficult for patients to wear and operate it for a long time. Researchers have investigated various control methods for hybrid systems of FES and exoskeletons to track the desired joint movements (Durfee, 2006; Quintero et al., 2010; Farris et al., 2011; Sharma et al., 2013; Ha et al., 2015; Chang et al., 2017). Ha et al. (2015) proposed to control the profile for muscle stimulation based on the difference between motor torque and muscle torque estimation to minimize the contribution of motor in joint trajectory tracking. Chang et al. (2017) developed a hybrid system for paraplegic patients utilizing a sensor-driven finite state machine to determine gait phases for stepping. Sharma et al. (2013) developed a gait simulation model of a hybrid system of FES and knee-ankle foot orthosis (KAFO), and by using the simulation model they proposed control parameters to optimize the gait rehabilitation system.

A challenging issue in controlling hybrid systems is the redundancy of actuation. Hybrid systems of the FES and exoskeleton inevitably have more actuators (biological muscles and artificial motors) than the degrees of freedom of the system. Studies have attempted to resolve the redundancy problem by using optimization methods to minimize the energy consumption of the actuators (Alibeji et al., 2017; Kirsch et al., 2017). Other approaches have utilized muscle synergy to reduce the dimensionality of actuation control (Alibeji et al., 2015; Li et al., 2019). However, these studies did not consider the volitional muscle activity of the patient, which adds complexity to the actuation control.

EMG is conventionally used to measure the muscle activity. Many studies have employed EMG to trigger and control the motion of exoskeletons (Frigo et al., 2000; Del-Ama et al., 2014; Bong et al., 2020; Yin et al., 2020). Del-Ama et al. (2014) proposed an EMG-based control system that is capable of maintaining motion-tracking performance by compensating for muscle fatigue. Yin et al. (2020) proposed to control the gait speed of the exoskeleton based on gait cycle duration extracted from surface EMG. When FES is applied in conjunction with EMG measurements, its electrical signals induce artifacts in the EMG signal. To extract the motion intention of the patient, the volitional EMG signal must be acquired from the raw EMG data by using artifact removal filters (Frigo et al., 2000; Bong et al., 2020). Recently, machine-learning techniques have been introduced to estimate the volitional EMG and predict the volitional joint torques generated by patients (Langzam et al., 2006; Zhou et al., 2020). Because volitional EMG signals indicate the patient’s involvement in joint motions, they can be used to coordinate the amplitude of the FES without excessive electrical stimulation of the muscles.

In this study, we developed a novel hybrid system of PAFO and FES along with its coordination control for gait rehabilitation according to the degree of volitional participation and stage of motor recovery of patients with post-stroke hemiplegia. The reference inputs supplied to the PAFO and FES were predetermined based on the gait data of healthy individuals. The PAFO was controlled to follow the reference joint angle and impedance trajectories, which were determined from biomechanical simulations. The reference input to the FES was derived from the EMG data of multiple individuals walking on a treadmill.

The CNN-based controller developed in this study coordinates the contributions of the PAFO and FES to generate the joint torque, which complements the torque generated by the volitional muscle activity of the patient. The effectiveness of the system was evaluated by using the data collected from individuals walking on a treadmill. The results showed that the system has the potential to improve the outcome of the rehabilitation program by inducing the active participation of the individual and providing assist-as-needed rehabilitation. By using the proposed system, the patient can be actively involved in the rehabilitation exercises, which can be conducted in an effective and timely manner depending on the recovery phase.

The remainder of this paper is organized as follows. Section 2 introduces the PAFO and FES of the hybrid system and its control methods. Section 3 presents a method for coordinating the contributions of the PAFO and FES based on the volitional EMG of the patient. Section 4 describes the experimental setup and results, followed by a discussion of the results. Finally, Section 5 presents the concluding remarks.

## 2 Hybrid system of PAFO and FES

**FIGURE 1:**
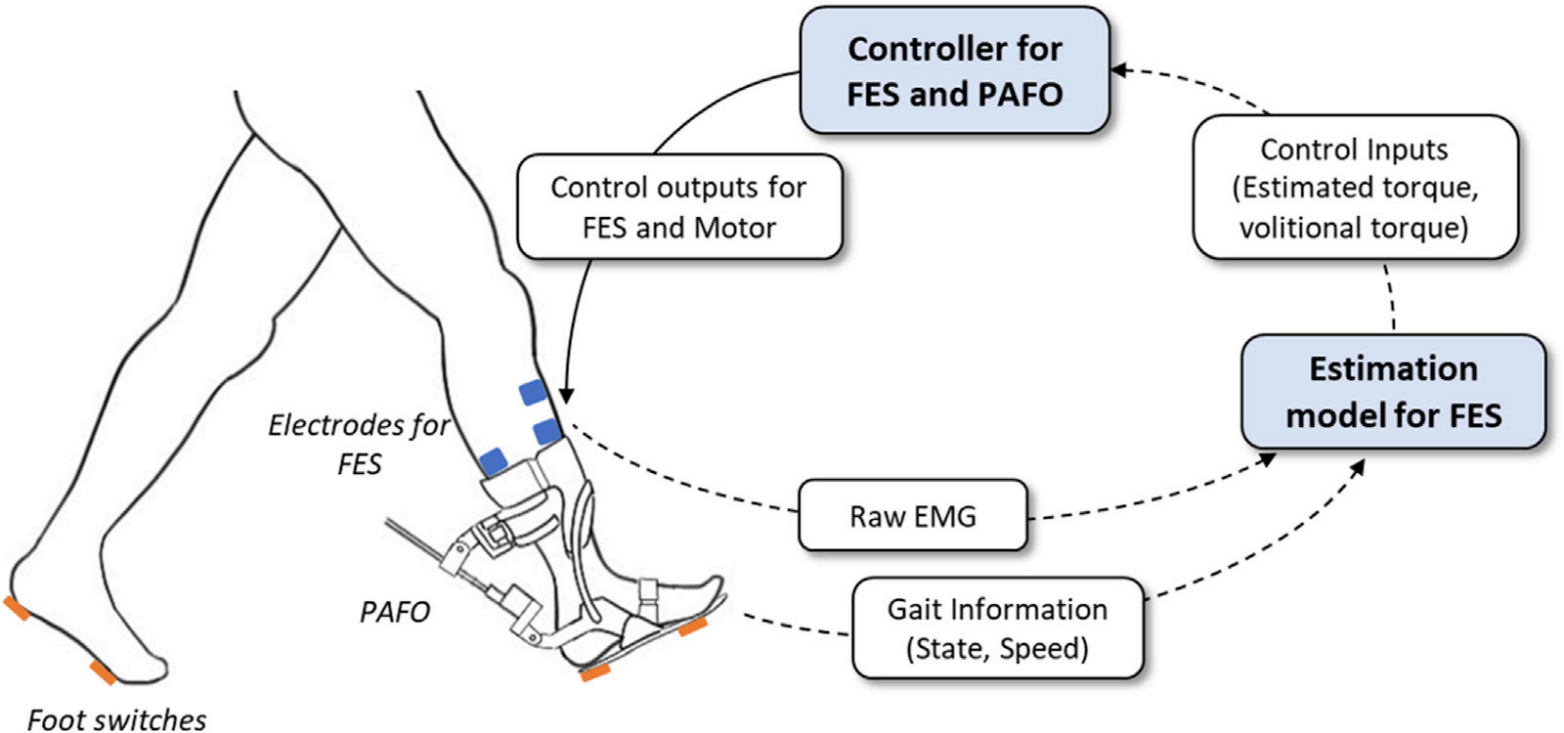
System overview of the hybrid gait rehabilitation system. The intensity of FES and desired trajectories of PAFO are determined based on the real-time estimation of the gait status and volitional torque.

FES and electrical motors provide effective assistance and rehabilitation for stroke survivors. Recent studies have attempted to integrate FES and motor-based orthoses to improve walking owing to the synergy of the two systems. In this study, we developed a hybrid PAFO and FES system for patients with hemiplegia, along with its control algorithm. Figure 1 presents an overview of the hybrid system. The developed system is composed of a PAFO equipped with a motor and foot switches in addition to electrodes for EMG and FES. An onboard controller was implemented in the PAFO to coordinate the contributions of the PAFO and FES in generating the joint torque.

### 2.1 PAFO control system based on impedance control

**FIGURE 2:**
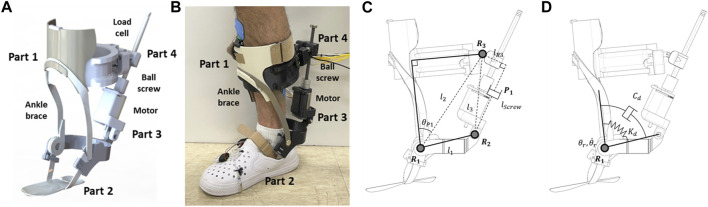
Schematic of 1-DOF PAFO. **(A)** 3D CAD model; **(B)** A person wearing the PAFO; **(C)** Kinematic diagram; **(D)** Dynamic modeling of ankle joint.

Figure 2 illustrates the PAFO developed in this study. As shown in the figure, it is composed of four parts: Part 1 is the upper part of the system, which is fastened tightly to the shank and equipped with a load cell. Part 2, the lower part of the system, has a rigid structure that transmits the motor torque to the ankle. Part 3 contains a brushless direct current (BLDC) motor (EC-max 40, Maxon Motor, Switzerland) and is connected to Part 2. Part 4 has a ball nut and is connected to Part 1. Parts 3 and 4 are connected with a ball screw. The PAFO has three revolute joints (R1-3) and one prismatic joint (P1), which allow ankle motion with one degree of freedom (DOF). To actuate the ankle joint, the BLDC motor generates a translational force through the ball screw with a diameter of 10 mm and lead of 2 mm. This mechanism enables the maximum torque of 0.4 Nm/kg, which can complement the joint torque generated by the muscles. A rotary encoder (AMT102-V, CUI Devices, United States) is placed on the shaft of the motor to measure the prismatic displacement of the ball screw. The interaction force between the shank and PAFO was measured using a load cell (CBFS-30, BONGSHIN LOADCELL Co., Ltd., Korea) embedded in Part 1. Four force-sensitive resistors (FSRs) were placed on the shoe soles to detect the contact between the heel, toe, and ground. The gait phase was estimated using threshold-based detection of the contact information from the FSRs. We modified an off-the-shelf ankle orthosis (Pacific Supply, Japan) for use as the frame of the PAFO.

The 1-DOF joint motion of the PAFO was controlled based on the kinematics and dynamics models illustrated in Figures 2C,D. The angle θ at the ankle joint (R1) can be expressed as the following kinematic equation using the parameters shown in Figure 2C.

$$\theta =\cos^{-1}\left(\frac{{l_{1}}^{2}+{l_{2}}^{2}-{l_{3}}^{2}}{2l_{1}l_{2}}\right)+\theta_{P1}=\cos^{-1}\left(\frac{{l_{1}}^{2}+{l_{2}}^{2}-{l_{screw}}^{2}-{l_{R3}}^{2}}{2l_{1}l_{2}}\right)+\theta_{P1}$$ (1)

Here, $l_{1}$ , $l_{2}$ , and $l_{3}$ denote the distances between joints R1-R2, R1-R3, and R2-R3, respectively. $l_{3}$ can be calculated from the moment arm of the joint R3 ( $l_{R3}$ ) and the distance between the ball nut and joint R2 ( $\left.l_{screw}\right)$ . $\theta_{P1}$ is a fixed angle determined by the structure of Part 1.

The dynamics model of the PAFO is described as follows:

$$I\ddot{\theta }=\tau_{A}+\tau_{ext}+\tau_{g}.$$ (2)

Here, I denotes the moment of inertia of the PAFO, and $\tau_{A}$ , $\tau_{ext}$ , and $\tau_{g}$ denote the torques generated by the actuator, external force, and gravity, respectively. The external torque $\tau_{ext}$ is calculated by using the interaction force between the shank and PAFO that is measured by the load cell as follows:

$$\tau_{ext}={F_{sensor}∙l}_{1}∙\sin\;\left(\cos^{-1}\left(\frac{{l_{2}}^{2}+{l_{3}}^{2}-{l_{1}}^{2}}{2l_{2}l_{3}}\right)+\tan^{-1}\left(\frac{l_{Screw}}{l_{R3}}\right)-\theta_{P1}\right)∙\cos\;\left({\cos^{-1}\left(\frac{{l_{1}}^{2}+{l_{3}}^{2}-{l_{2}}^{2}}{2l_{1}l_{3}}\right)+\tan}^{-1}\left(\frac{l_{R3}}{l_{Screw}}\right)-\frac{\pi }{2}\right)$$ (3)

where $F_{sensor}$ is the force measured by the load cell.

The control law to generate the actuator torque is as follows:

$$\tau_{A}:=\tau_{c}-{\hat{\tau }}_{g}.$$ (4)

where $\tau_{c}$ and ${\hat{\tau }}_{g}$ denote the torque generated by impedance control and the torque compensation for gravity, respectively.

Figure 2D shows the desired stiffness $k_{d}$ and damping $c_{d}$ of the impedance control to ensure soft contact of the PAFO with the rigid ground. An impedance controller was implemented to generate the torque as follows:

$$\tau_{c}=-\left(k_{d}e+c_{d}\dot{e}\right)$$ (5)

where e denotes the error between the measured joint angle θ and reference joint angle $\theta_{Ref}$ ( $e=\theta -\theta_{Ref}$ ). The reference joint trajectory ( $\theta_{ref}\left(t\right)$ ) and desired impedance ( $k_{d}$ and $c_{d}$ ) were predetermined using the methods described in Sections 3.1 and 3.3, respectively.

### 2.2 FES control system based on 1D CNN estimation model

**FIGURE 3:**
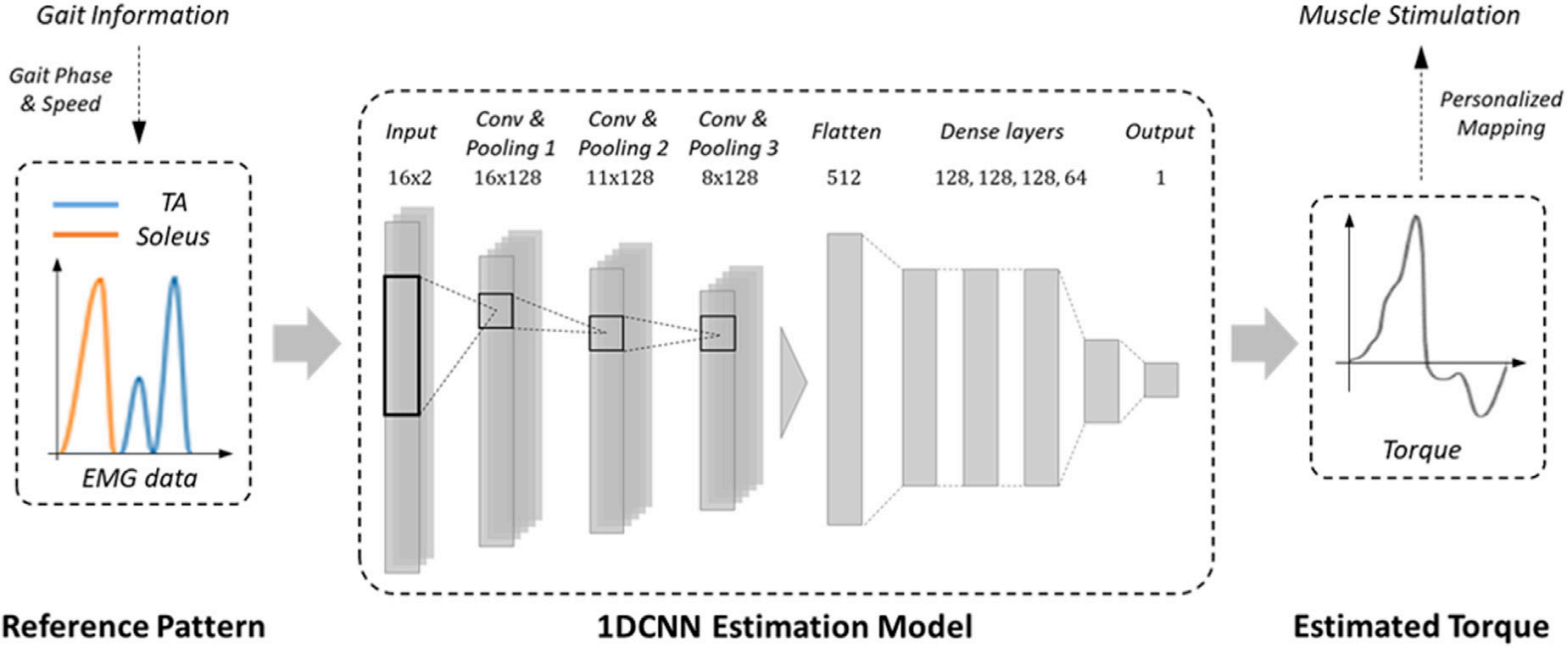
Schematic view of FES control algorithm.

The FES was controlled using an algorithm developed in our previous study (Jung et al., 2021). A two-channel FES system stimulates the tibialis anterior (TA) and soleus muscles for ankle dorsiflexion and plantar flexion during walking. Figure 3 shows the configuration of the control system. The control algorithm computes the FES stimulation patterns corresponding to various gait speeds. The gait phase and speed were derived from the contact information of the FSRs attached to the heels and toes of the shoe soles to determine the parameters for one gait cycle.

In this study, we followed four steps to reconstruct the EMG profiles of the TA and soleus muscles adaptive to the walking speed: 1) acquisition of EMG data on the treadmill at walking speed ranging from 1 to 5 km/h with six healthy individuals, 2) construction of normalized profiles for five representative walking speeds (1,2,3,4 and 5 km/h) during one gait cycle, 3) formulation of equations via curve fitting of the five profiles composed of trigonometric functions, 4) modulation of the parameters (phase shift, period, and amplitude) of the trigonometric function depending on the walking speed. Based on the gait speed, the resulting function produces reference EMG patterns for the TA and soleus of one gait cycle in real-time.

A regression model based on a one-dimensional convolution neural network (1D CNN) is used to obtain the reference ankle torque from the two-channel reference EMG patterns as shown in Figure 3. The architecture of the suggested model includes three convolution layers, each followed by a pooling layer. The convolution layers had a filter size of 128, while the pooling layers had sizes of 6, 4, and 2. Each kernel in the CNN layer has sizes of 13, 9, and 6, and processes the data with a stride of 1 using rectified linear unit (ReLU) as the activation function. The output data from the CNN layers is then resized by a flattened layer, and transmitted to the MLP. A series of five MLPs is connected with layer sizes of 129, 128, 128, 64, and 1. For the activation functions for the first four MLPs, rectified linear unit (ReLU) are used, and for the last MLP, hyperbolic tangent function (Tanh) is to reflect the data’s nonlinearity. The regression model was trained using the Adam optimizer, and the loss was calculated through mean squared error (MSE). By monitoring the validation loss, the training was finished after reaching a certain level of accuracy to avoid overfitting of the model.

The neural network model was trained by using experimental data acquired from isometric contraction tests of the ankle. From six healthy subjects, EMG data from the TA and soleus along with the ankle joint torque was measured, when external force was applied in the plantar flexion (positive torque) and isometric dorsiflexion (negative torque) directions. The collected data was converted into time series data with two channels of EMG data and one channel of joint torque data. The data was divided into training, evaluation, and test datasets with a ratio of 6:2:2. The trained 1D CNN model showed the root mean square error (RMSE) of 4.97 and the peak accuracy of 91.17% by using the test dataset. By giving the reference patterns as the input to the 1D CNN model, the corresponding ankle joint torque is estimated to generate the gait motion in accordance with the speed and phase of the gait. The intensity and duration of the FES are then modulated based on the estimated joint torque and personalized parameters of the patient, as described in Section 3.3.

## 3 Coordination control of PAFO and FES

**FIGURE 4:**
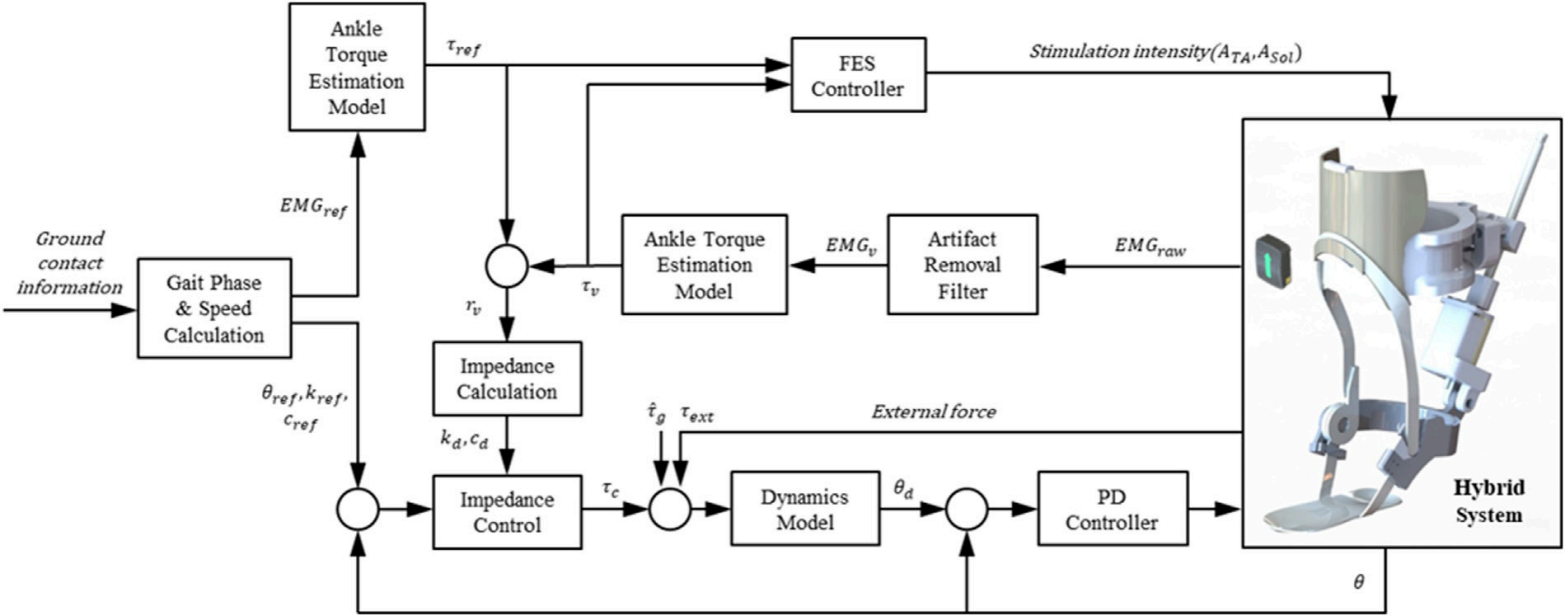
Control scheme of hybrid system of PAFO and FES. The main loop initializes the reference inputs using the gait phase and speed calculated from the ground contact event. The FES, PAFO, and volitional torque generated by each sub-system are used to calculate the ratio of each sub-system. Finally, a reduced output of PAFO and FES is generated depending on the volitional torque value.

This section presents a control method for coordinating the contributions of the PAFO and FES to produce the ankle joint torque. Figure 4 illustrates the control scheme for the PAFO and FES hybrid system. Based on the gait phase and speed estimated from the ground contact events, reference inputs acquired from biomechanical model simulations were supplied to the PAFO and FES controllers. The reference inputs to the FES control were obtained from the EMG data recorded from healthy individuals walking on a treadmill. To coordinate the contributions of the PAFO and FES, the torque generated by the volitional muscle activity needs to be considered. The volitional joint torque is estimated from the volitional component of the EMG using a 1D CNN model (Jung et al., 2021). The joint torque produced by the PAFO and FES complements the torque generated by the volitional muscle activity. Feedback control is used to correct the position error between the actual joint angle and desired joint angle estimated from the dynamics model of the PAFO described in Eq. 2.

### 3.1 Preparation of reference inputs to PAFO and FES

A biomechanical simulation was performed to obtain the reference inputs for the PAFO. The ankle movement during gait was analyzed using a musculoskeletal model in the OpenSim software (SimTK, Stanford, CA) (Delp et al., 2007). This software was used to simulate the gait motion of a human model reflecting increased weight and inertia by a PAFO (Jung et al., 2021). The human musculoskeletal model was based on the 23-DOF Gait 2,354 model composed of the lower extremities and torso. To account for the coupled dynamics with the human musculoskeletal model, a multi-body dynamics model of the PAFO was added. By kinematic and dynamic simulations, the reference joint angle and torque were obtained to be used as control inputs to PAFO.

**FIGURE 5:**
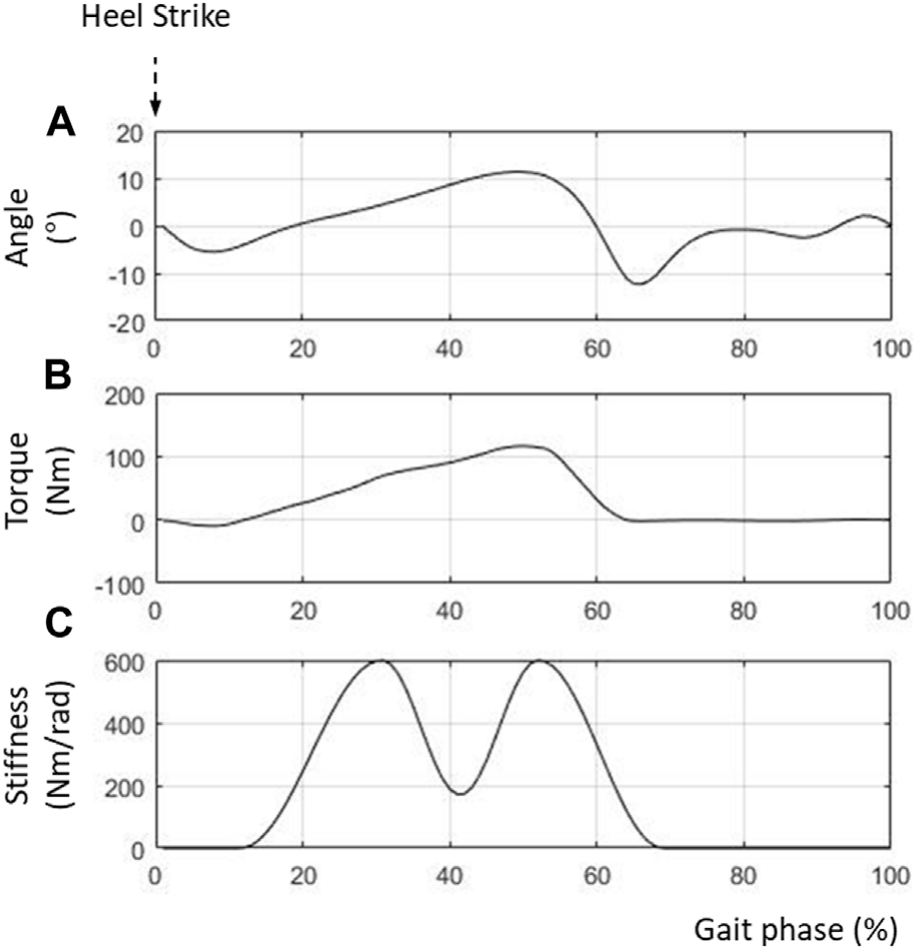
Control inputs for PAFO. **(A)** Reference ankle angle; **(B)** Reference joint torque; **(C)** Reference joint stiffness.

Figure 5 shows the simulation results for the human model wearing the PAFO. Figures 5A,B show the reference angles ( $\left.\theta_{ref}\right)$ and torque at the R1 joint of the PAFO, respectively. The reference joint stiffness $k_{ref}$ is estimated by determining the slope of the moment-angle curve constructed from Figures 5A,B (Kern et al., 2019). As shown in Figure 5C, the stiffness curve exhibited two peaks during the gait cycle. The peak at around 30% gait cycle indicates the strong support of the ankle joint in the loading response, whereas the other at around 50% gait cycle indicates the powerful propulsion of the ankle joint.

**FIGURE 6:**
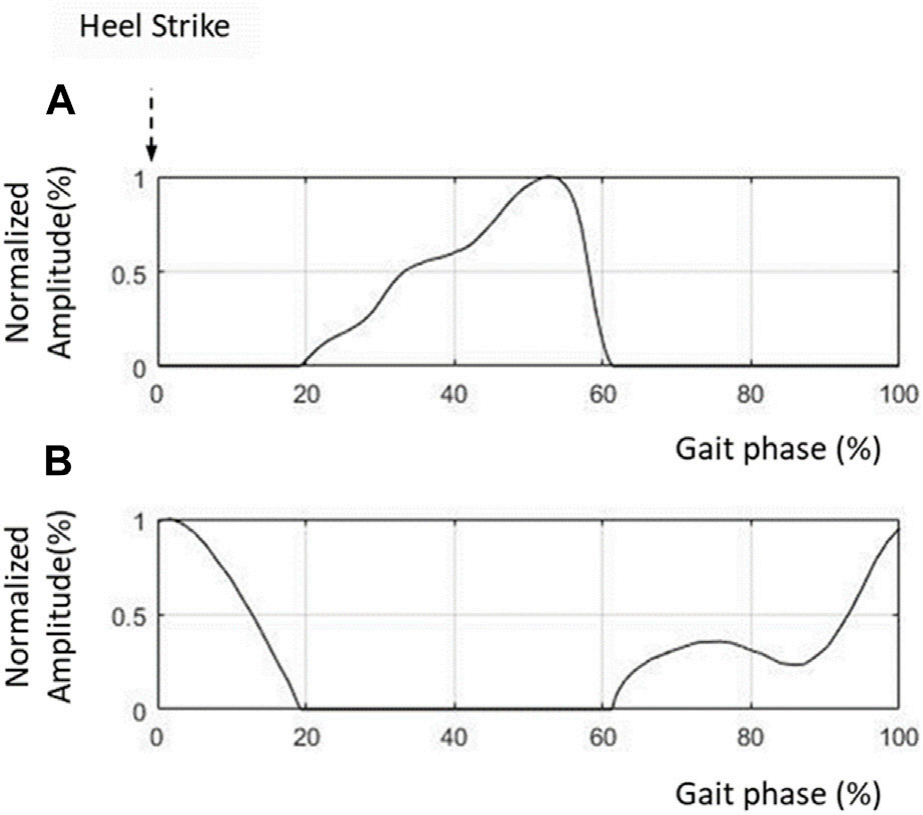
Control inputs to FES. **(A)** FES amplitude for TA; **(B)** FES amplitude for soleus.

The reference input to the FES was created based on the EMG data collected from healthy individuals walking on a treadmill. After averaging and normalizing the EMG data from the TA and soleus, the envelopes of the EMG patterns were functionalized using the parameters for various gait speeds. Figures 6A,B show the reference inputs used to stimulate the TA and soleus, respectively. Figure 6A shows the muscle activity in the soleus for generating propulsion in the stance phase (20%–60% gait cycle). Figure 6B shows the required muscle activities in the TA from the swing phase (60%–100% gait cycle) to the heel strike (0%–20% gait cycle).

### 3.2 Consideration of volitional muscle activity

In FES control, the volitional muscle activity of the patient should be considered to apply the correct amount of stimulation to the muscle to generate the desired movement. The rehabilitation of post-stroke hemiplegic lower limbs aims to improve the volitional ability to activate the leg muscles. However, the active participation of the patient in the rehabilitation is essential. Depending on the patient’s stage of motor recovery, the amount of FES should be controlled to complement the volitional muscle activity in generating the desired movement. This prevents excessive muscle stimulation and fatigue.

Volitional muscle activity can be measured using EMG by filtering out artifacts that appear when FES and EMG are applied to the same muscle. Several studies have suggested the use of calculation-based filters to remove artifacts (Langzam et al., 2006; Erez et al., 2010; Bong et al., 2020). These studies investigated methods for suppressing large artifacts induced by FES. After filtering out large FES artifacts, the M-waves are filtered to obtain a pure volitional EMG.

**FIGURE7:**
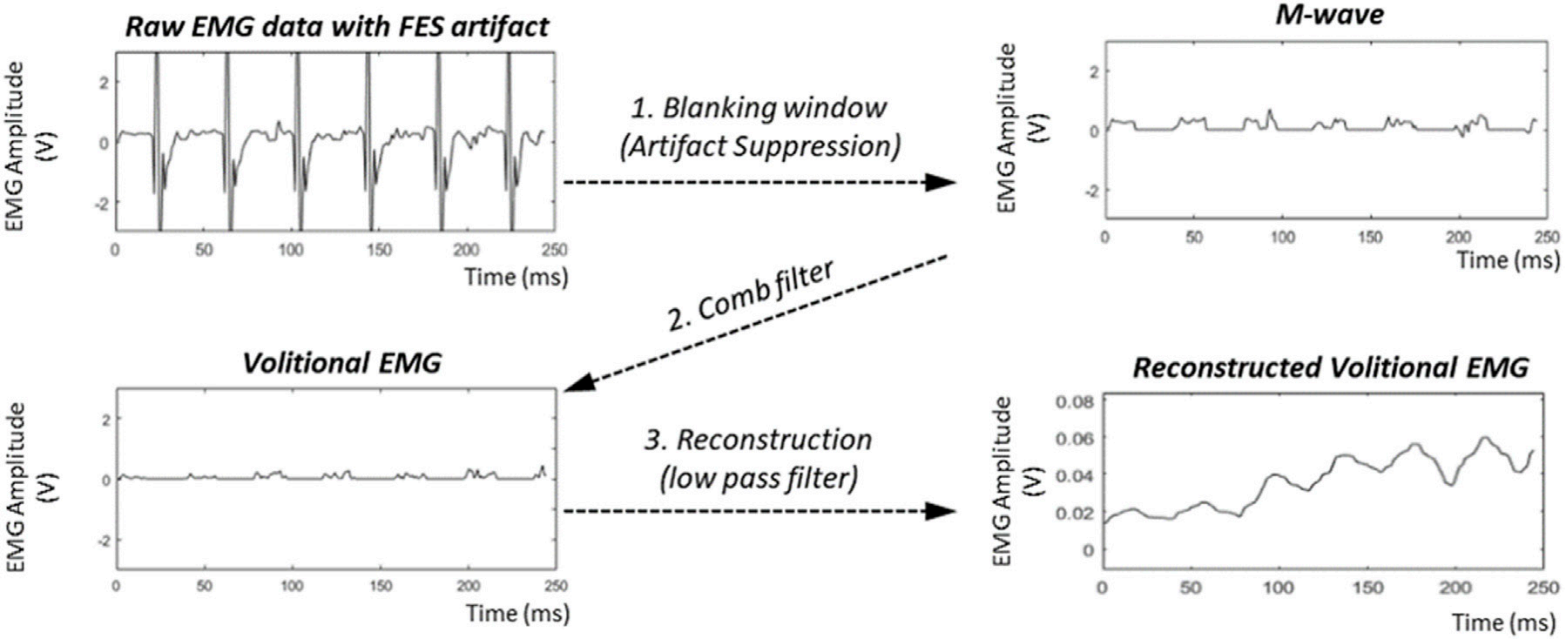
Filtering process to obtain volitional EMG.

In this study, we selected a series of filters to rectify the raw EMG data because of its simplicity and effectiveness in real-time applications. Figure 7 shows the three steps used to obtain the volitional EMG. First, the blanking window removes the artifacts in each pulse of the FES, which may overwhelm the M-wave. By eliminating the FES artifacts using a blanking window, a mixture of volitional EMG and M-wave EMG can be observed. Second, the comb filter computes the volitional EMG using the following equation:

$$y\left(t_{i}\right)=\left(x\left(t_{i}\right)-x\left(t_{i-1}\right)\right)/\sqrt{2},$$ (6)

where $x\left(t_{i}\right)$ and $x\left(t_{i-1}\right)$ denote the M-waves from the current and one-step previous FES pulses, respectively, and $y\left(t_{i}\right)$ denotes the current volitional EMG. The final step yields the reconstructed volitional EMG using a low-pass filter with a cut-off frequency of 2Hz to compensate for the discontinuity caused by the blanking window used in the first step.

The extracted volitional EMG is then converted into volitional joint torque using the EMG torque estimation model described in Section 2.2.

**FIGURE 8:**
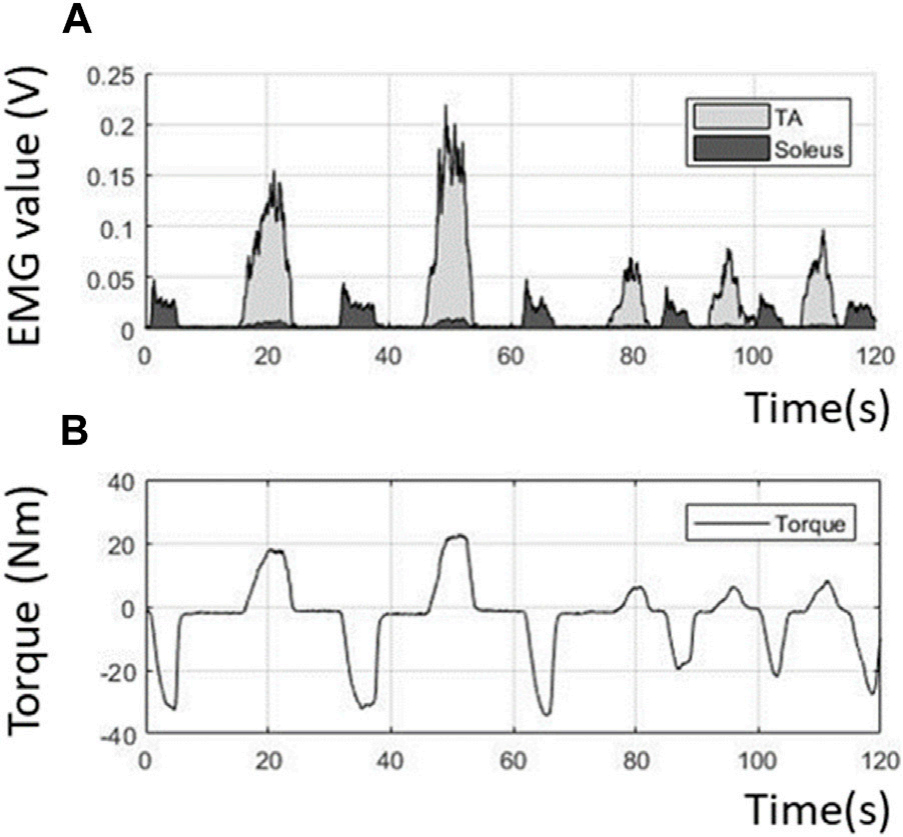
Volitional EMG and torque obtained from the filtering process. **(A)** Extracted volitional EMG; **(B)** Estimated volitional torque from the EMG.

Figure 8 shows the volitional EMG and volitional joint torque estimated from the voluntary contraction of the TA and soleus during dorsiflexion and plantarflexion. The ratio of the contributions of the PAFO and FES is determined based on the volitional joint torque.

### 3.3 Coordination of PAFO and FES

**FIGURE 9:**
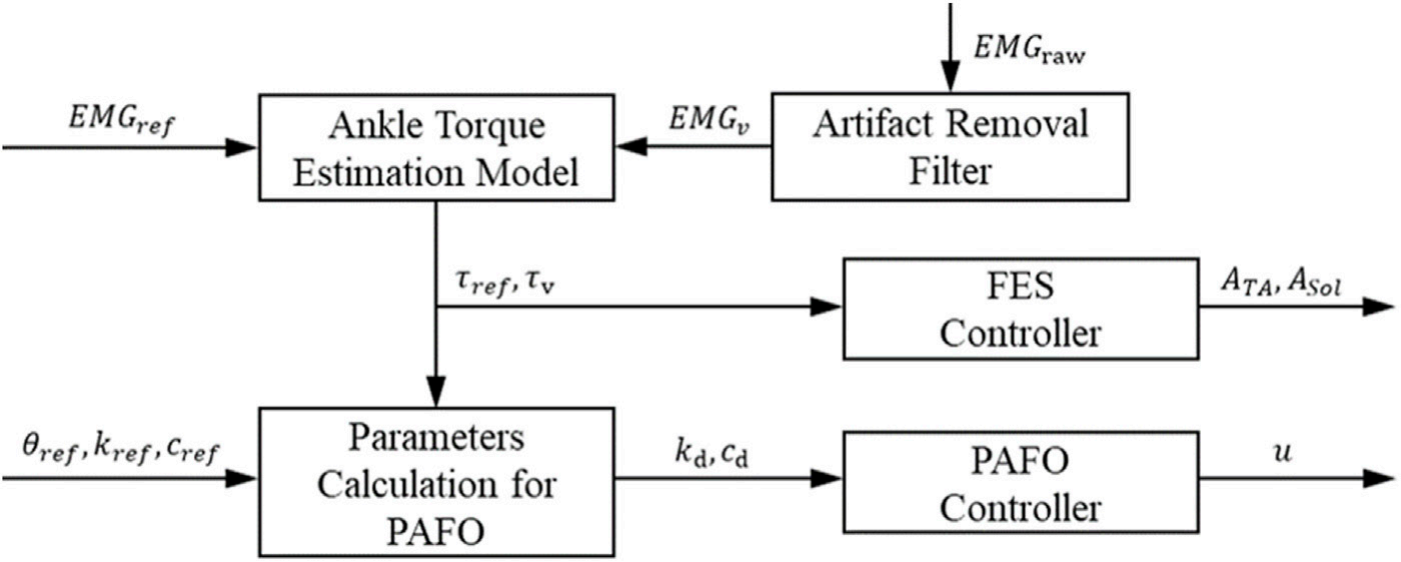
Sub-system for coordination control of ratio between torques generated by PAFO and FES.

Figure 9 illustrates the control scheme used to coordinate the contributions of the PAFO and FES in generating the joint torque. As described in Section 3.1, the reference inputs to the PAFO and FES were the reference joint angle, stiffness, damping ( $\left.\theta_{ref},k_{ref},c_{ref}\right)$ , and reference EMG pattern ( $\left.{EMG}_{ref}\right)$ . With the reference EMG input ${EMG}_{ref}$ , the reference joint torque $\tau_{ref}$ was computed using the EMG torque estimation model described in Section 2.2.

The relationship between the FES intensity and the corresponding torque was assumed to be linear, as in the study by Ito et al. (2007). Personalized parameters for the linear relationships were determined using a simple FES sensitivity test to determine the onset point and pain threshold of the TA and soleus in each participant (Jung et al., 2021). The TA and soleus were assumed to be activated without co-contraction during gait, and the amplitudes of the FES supplied to the TA and soleus were proportional to the negative and positive reference joint torques, respectively (Ito et al., 2007).

After estimating the volitional torque $\tau_{v}$ from the extracted volitional EMG, as described in Section 3.2, the FES intensities for the TA and soleus ( $A_{TA},A_{Sol}$ ) were determined as follows:

$$A_{TA}=\left(A_{TA,\max}-A_{TA,\min}\right)∙{\left(\frac{\tau_{ref}-\tau_{v}}{\tau_{ref,\max}}\right)}_{-}+A_{TA,\min},$$ (7)

$$A_{Sol}=\left(A_{Sol,\max}-A_{sol,\min}\right)∙{\left(\frac{\tau_{ref}-\tau_{v}}{\tau_{ref,\max}}\right)}_{+}+A_{sol,\min},$$ (8)

where the FES intensities $A_{TA}$ and $A_{Sol}$ were controlled within the personalized range between $A_{TA,\max}$ and $A_{TA,\min}$ and between $A_{Sol,\max}$ and $A_{sol,\min}$ for the TA and soleus, respectively, to prevent excessive application of stimulation. The term $\left(\tau_{ref}-\;\tau_{v}\right)/\tau_{ref,\max}$ reflects the ratio between the joint torques produced by the FES-induced and volitional muscle activities. If the volitional torque exceeds the reference torque ( $\tau_{v}>\tau_{ref}$ ), the FES controller generates the minimum required amplitudes to generate the torque ( $\left.A_{TA,\min},A_{Sol,\min}\right)$ . If no volitional torque is estimated ( $\tau_{v}=0$ ), the FES controller generates amplitudes that follow the same profile as the reference torque ( $\left.\tau_{ref}\right)$ . The negative and positive signs in Eqs 7, 8 indicate that the torques generated by the TA and soleus contribute to dorsiflexion (negative torque) and plantar flexion (positive torque), respectively.

Using the reference torque $\tau_{ref}$ and volitional torque $\tau_{v}$ , the activation ratio $r_{v}$ between the volitional muscle activity and reference muscle activation is determined as follows:

$$r_{v}=\frac{\text{ma}x\left(\begin{array}{cc} \tau_{ref}, & \tau_{v} \end{array}\right)}{\tau_{v,\max}},$$ (9)

where $\tau_{v,\max}$ is the maximum torque generated by the volitional muscle activation. The ratio $r_{v}$ has a value between 0 and 1, where $r_{v}=0$ indicates that the PAFO solely contributes to the joint torque, and $r_{v}=1$ indicates the maximum contribution of the volitional muscle activity to the joint torque. Equation 9 indicates the contribution of volitional muscle activities for most healthy individuals and chronic patients for whom volitional torque $\tau_{v}$ is greater than reference torque $\tau_{ref}$ . For the patient who is unable to produce muscle contraction on their own ( $\tau_{ref}$ is greater than $\tau_{v}$ ), however, $r_{v}$ is set to 0 so that the gait can be fully assisted by PAFO and FES.

Using the activation ratio $r_{v}$ , the desired joint stiffness $k_{d}$ of the PAFO is determined as follows:

$$k_{d}=k_{\max}∙\left(k_{ref}-{r_{v}\times k}_{ref,\max}\right)/k_{ref,\max}+k_{\min}\;.$$ (10)

Here, $k_{ref,\max}$ denotes the maximum value of the reference stiffness. The reference stiffness $k_{ref}$ was predetermined as described in Section 3.1. The minimum and maximum stiffness values were set between $k_{\max}$ and $k_{\min}$ to avoid excessive movement of the PAFO. The desired damping $c_{d}$ is set by using the following equation:

$$c_{d}=2\zeta \sqrt{k_{d}},$$ (11)

where ζ is the damping ratio.

As can be seen in Figure 4, the desired joint angle $\theta_{d}$ of the PAFO is determined by using dynamics model described in Eqs 1––5, and its parameters described in Eqs 10 and 11.

$$I{\ddot{\theta }}_{d}=-\left(k_{d}e+c_{d}\dot{e}\right)-{\hat{\tau }}_{g}+\tau_{ext}+\tau_{g}\approx -\left(k_{d}e+c_{d}\dot{e}\right)+\tau_{ext}$$ (12)

To follow the desired joint angle $\theta_{d}$ , a proportional derivative controller is used as follows:

$$u=K_{P}e_{d}+K_{D}{\dot{e}}_{d},$$ (13)

where $e_{d}$ denotes the error between the measured and desired angles ( $e_{d}=\theta -\theta_{d}$ ), and u , $K_{P}$ , and $K_{D}$ represent the control signal, proportional gain, and derivative gains, respectively. The controller gains were empirically determined.

## 4 Experimental setups and results

### 4.1 Experimental devices and protocol

**FIGURE 10:**
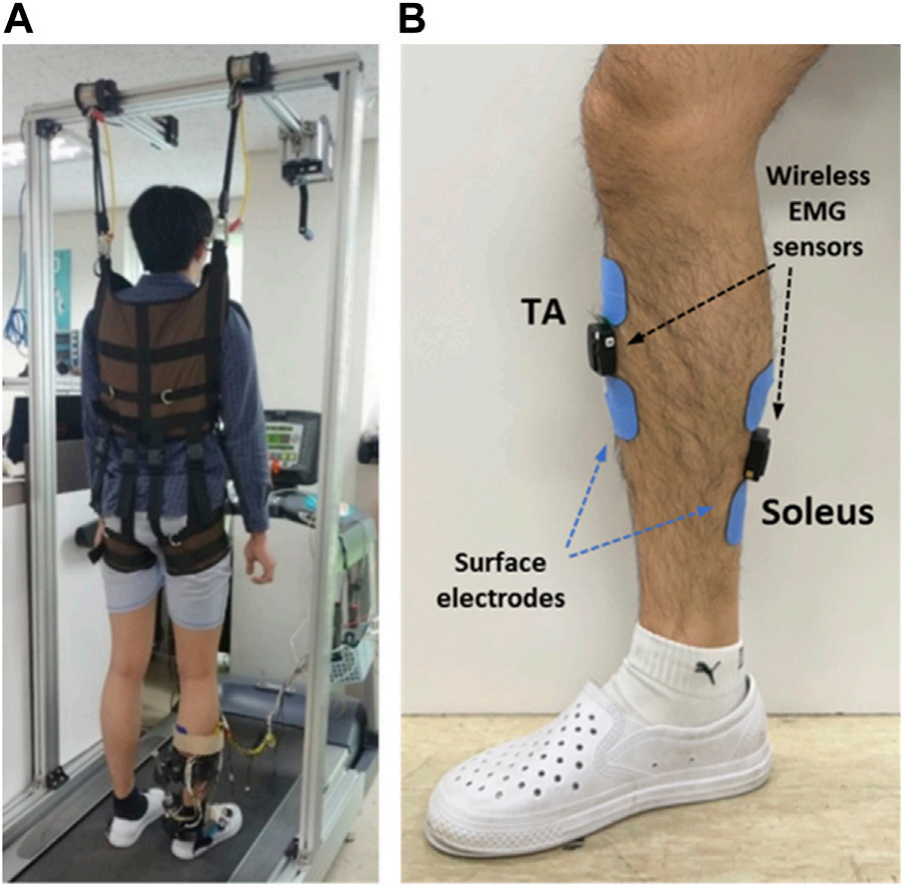
Experimental setup for treadmill walking. **(A)** Participant in position on the treadmill; **(B)** Placement of EMG sensors and FES electrodes.

Experiments were conducted with healthy participants walking on a treadmill to test the performance of the developed system. Figure 10A shows the experimental setup used to validate the developed hybrid system. Three healthy males in their twenties and thirties participated in this study. Prior to starting the experiment, the participants were fully informed about the experimental procedure and informed consent was obtained. All the experimental equipment and procedures were approved by the Deliberation Committee (KUIRB-2020-0277-01). The participants performed treadmill gait in two experimental modes: the PF mode (PAFO and FES without considering the volitional muscle activity of the patient) and PFV mode (PAFO and FES considering the volitional muscle activity of the patient). The participants were notified that they could terminate the stimulation at any time through an emergency switch if they experienced discomfort or any other abnormality. The experiment was limited to 1 hour, and the three participants began the experiments after they had sufficiently adapted to treadmill walking at a speed of 1.8 km/h.

Before the experiments, the FES pain threshold and maximum ankle torque of each participant were measured using simple isometric contraction tests. Based on these personalized parameters, the intensity of the FES and stiffness of the PAFO were tuned according to the gait phase. Figure 10B shows the placement of the EMG sensors and FES electrodes on a participant. Two wireless EMG sensors and two FES electrodes were attached to the shank skin. The EMG sensor for the TA was placed on the belly of the muscle and the sensor for the soleus was placed at the center of the muscle length located below the medial gastrocnemius to avoid unwanted involvement of the calcaneal tendon. After placement of the EMG and FES electrodes, the participant wore the PAFO, which was set to the neutral position of the ankle, and then put on the experimental shoes. For safety and guidance for gait motion, the participant was secured with a harness installed on the treadmill (Figure 10A). Considering safety during the experiment, the participants were instructed to hold the emergency stop switch to stop the test if needed.

After finishing the experiment, the participants were asked to respond to 3-item Likert questions regarding naturalness of gait motion, comfortableness of FES, and ease of muscle fatigue with the PF and PFV modes (Q1) The gait motion felt natural. (Q2) The electrical stimulation of FES felt comfortable without causing much pain (Q3) The gait experiment did not cause much muscle fatigue. A 5-point Likert scale was used in the questionnaire: (1) strongly disagree, (2) disagree, (3) neither agree or disagree, (4) agree, and (5) strongly agree.

### 4.2 Experimental results

**FIGURE 11:**
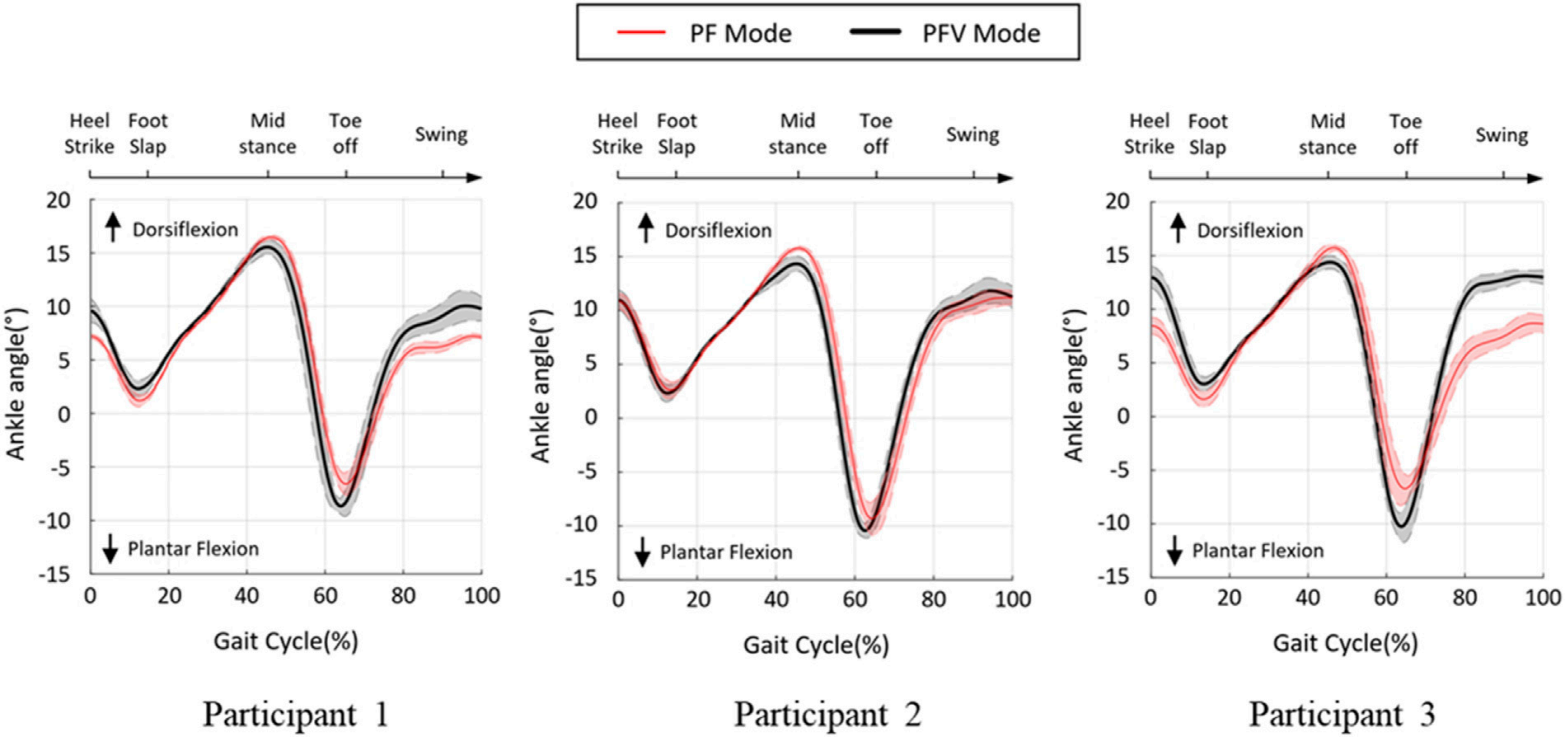
Ankle angle during treadmill gait. Black line and red line denote the PF and PFV modes, respectively.

During the gait cycle, the ankle angle was measured to compare the performances of the PF and PFV modes of the hybrid system. The ankle angle was calculated from the motor encoder data using the PAFO kinematics. Figure 11 shows the angle trajectories of the three participants during one gait cycle along with the error graphs. The range of motion (ROM) varied between −10° and 15° for all participants, with distinct patterns for the two modes. At approximately 15% gait cycle, the negative peaks in the direction of the plantar flexion were limited for both modes by providing sufficient dorsiflexion torque to avoid foot-slap caused by premature contact of the sole of the foot with the ground. For all participants, the maximum dorsiflexion at approximately 45% gait cycle during the midstance was smaller in the PFV mode than in the PF mode. In the PFV mode, dorsiflexion due to excessive FES was avoided during midstance. At toe-off (65% gait cycle), the PFV mode showed larger peaks in the direction of plantar flexion, generating more powerful propulsion than the PF mode. During the swing phase (80%–90% gait cycle), the PFV mode showed larger dorsiflexion than the PF mode. Increased dorsiflexion is advantageous in preventing foot drop and securing toe clearance during the swing phase.

**FIGURE 12:**
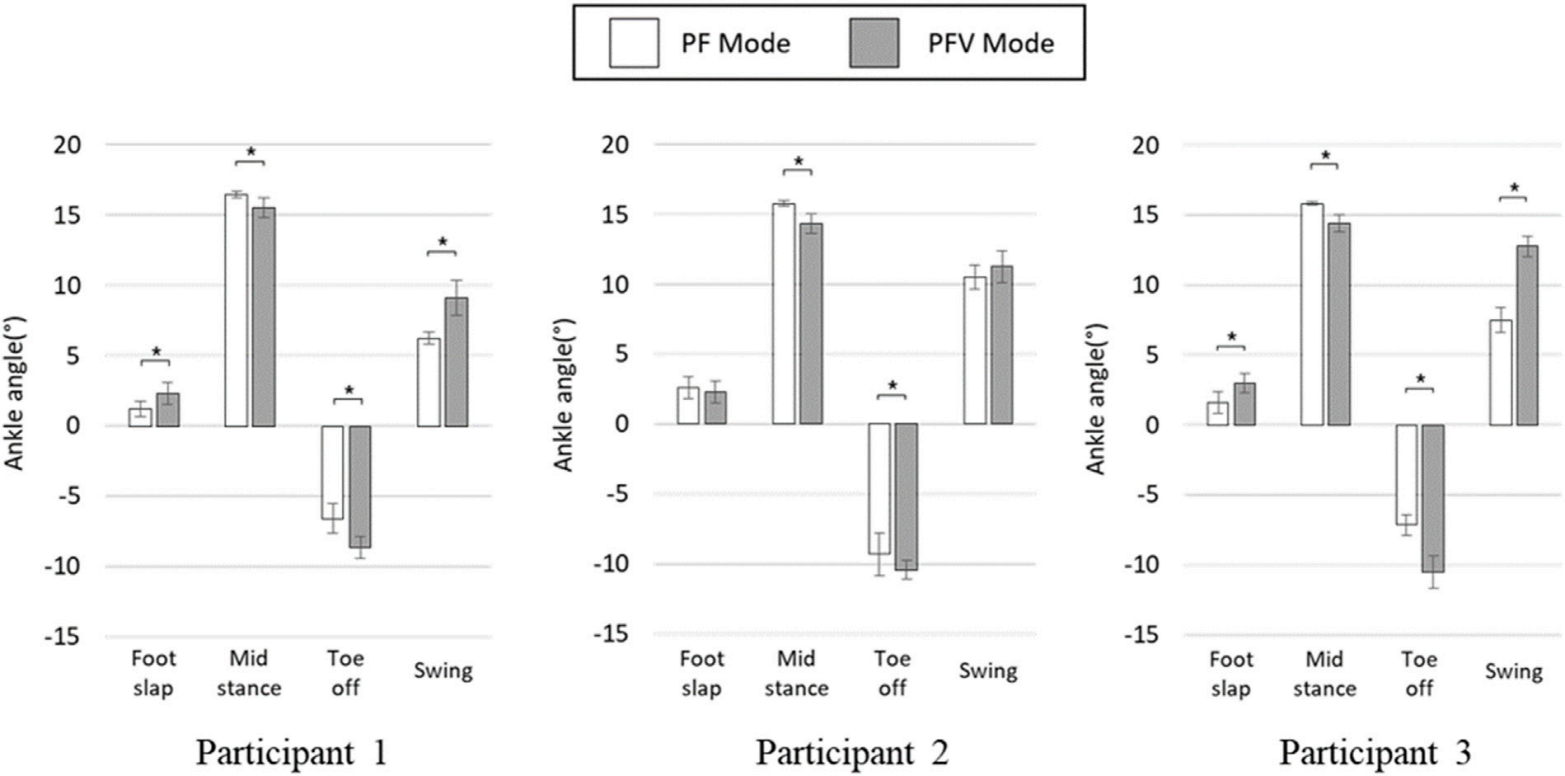
Four featured peaks of the ankle angle trajectories (* denotes *p*-value under 0.05).

Figure 12 compares the four featured peaks of the ankle angle trajectories at 15%, 45%, 65%, and 90% gait cycles, where the positive and negative values indicate dorsiflexion and plantar flexion of the ankle, respectively. As can be seen in the figure, there were statistically significant differences between the two modes at 45% and 65% gait cycles for all participants. However, at 15% and 90% gait cycles, the two modes appeared to show no distinct differences.

**FIGURE 13:**
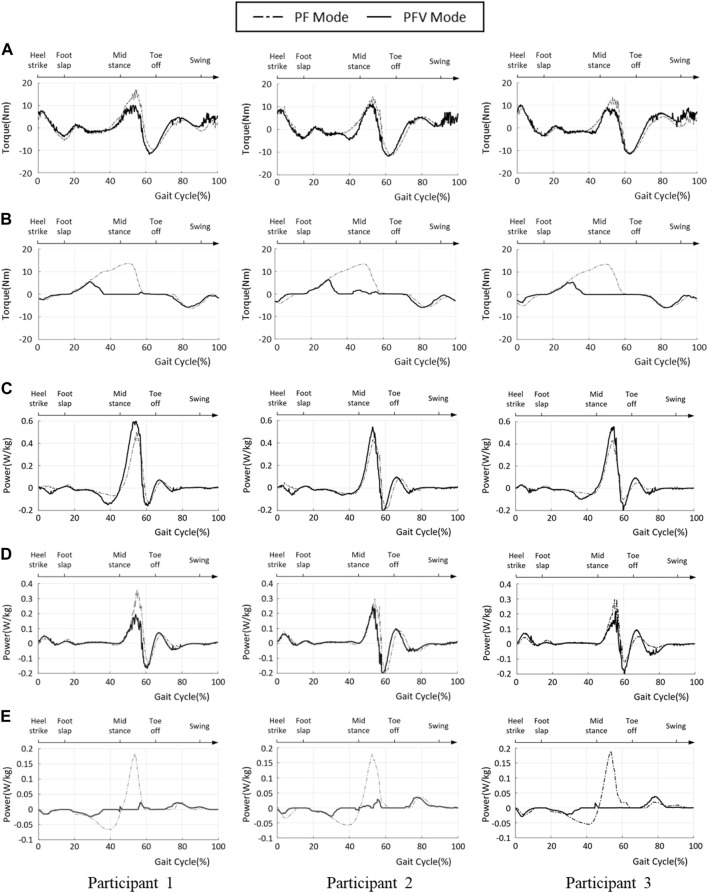
Ankle joint power of PF and PFV modes. **(A)** Torque generated by the PAFO; **(B)** Torque generated by the FES; **(C)** Total power generated by the hybrid system; **(D)** Power generated by PAFO; **(E)** Power generated by FES (solid line: PFV mode, dash-dotted line: PF mode).

To evaluate the effectiveness of gait assistance in the two modes, the ankle joint power generated by the PAFO and FES was computed by multiplying the torque and angular velocity at the ankle joint. The joint torque generated by the PAFO was computed based on its kinematics and control output, as described in Section 2.1, and the volitional and FES-induced joint torques were estimated using the 1D CNN model described in Section 2.2. Figures 13A–E depict the joint torque and power generated by the hybrid system, plotted using the ensemble averages of the gait cycles. By considering volitional muscle activities, the torque generated by motor and FES (shown as (A), (B) in Figure 13), is lower with the PFV mode than with the PF mode. In the PFV mode, the amplitude of FES is significantly suppressed to its minimum during the stance phase as the volitional muscle contraction becomes dominant (Figure 13B).

In the PF mode, the total power is the sum of the powers generated by the PAFO and FES. In the PFV mode, the total power is the sum of the power generated by the PAFO, FES, and voluntary muscle activities. The ankle joint powers in the PF and PFV modes are plotted as dashed and solid lines, respectively. As shown in Figure 13C, the total power generated in the PFV mode was higher than that in the PF mode for all participants. This is because the power generated by the volitional muscle activity is included in the total power generation in the PFV mode. However, the difference between the PF and PFV modes was small. Figure 13D shows that the PAFO generated less power in the PFV mode than in the PF mode for all participants. Figure 13E shows that the FES generates significantly lower power in the PFV mode than in the PF mode. Figures 13D,E show that the differences between the two modes are remarkable, particularly in the midstance phase (30%–60% gait cycle).

**FIGURE 14:**
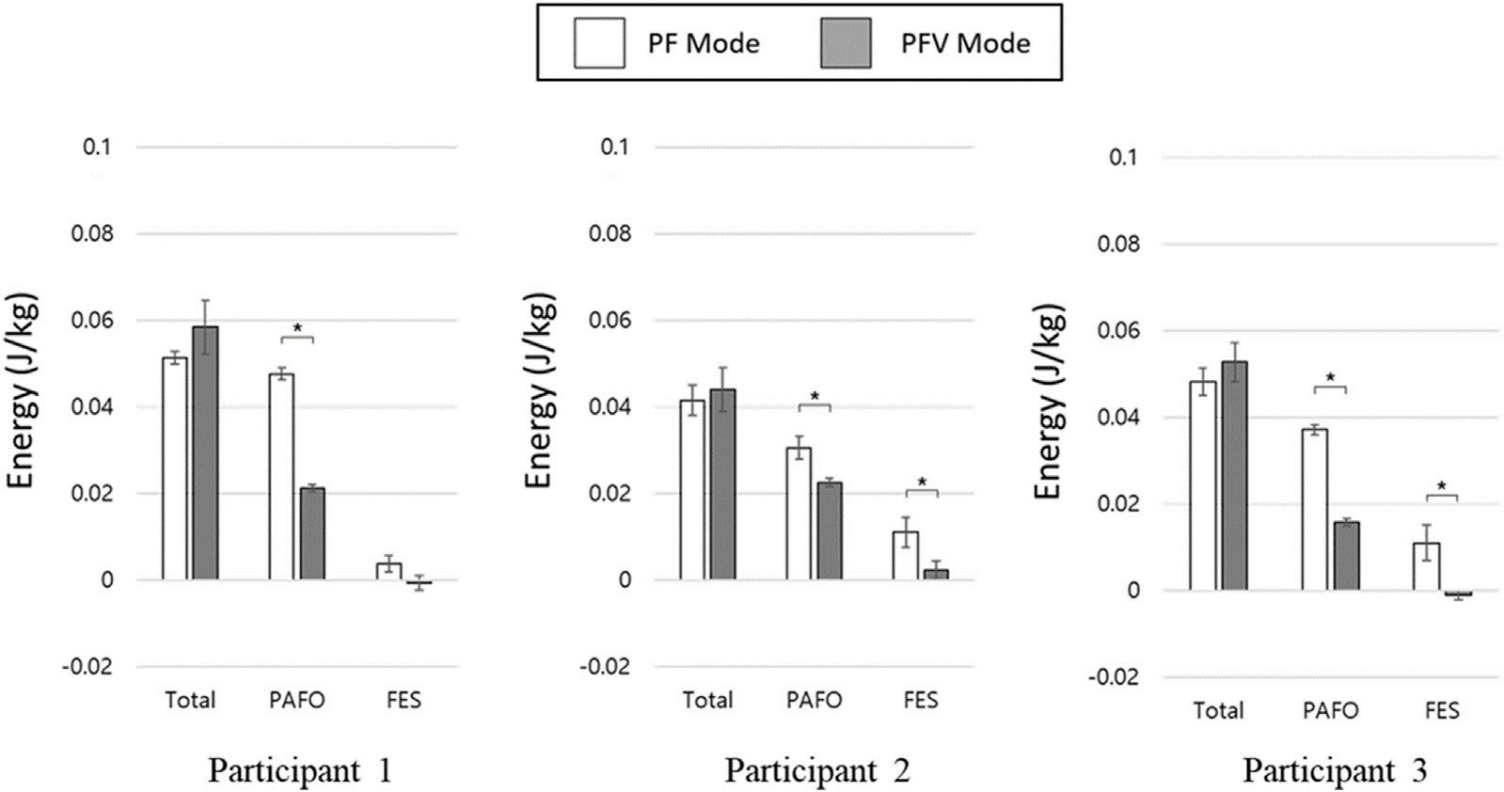
Ankle joint energy of PF and PFV modes. (* denotes *p*-value under 0.05).

Figure 14 shows a comparison of the ankle joint energy per gait cycle for the two modes. The ankle joint energy was computed using the time integral of the ankle joint power during one gait cycle. As shown in the figure, the total joint energy is higher in the PFV mode than in the PF mode, whereas the difference between the two modes is not statistically significant. The joint energies generated by the PAFO and FES were significantly higher in the PF mode than in the PFV mode. These results demonstrate that power consumption can be greatly reduced using the PFV mode when compared with the PF mode by taking advantage of the power generation by volitional muscle activities.

**FIGURE 15:**
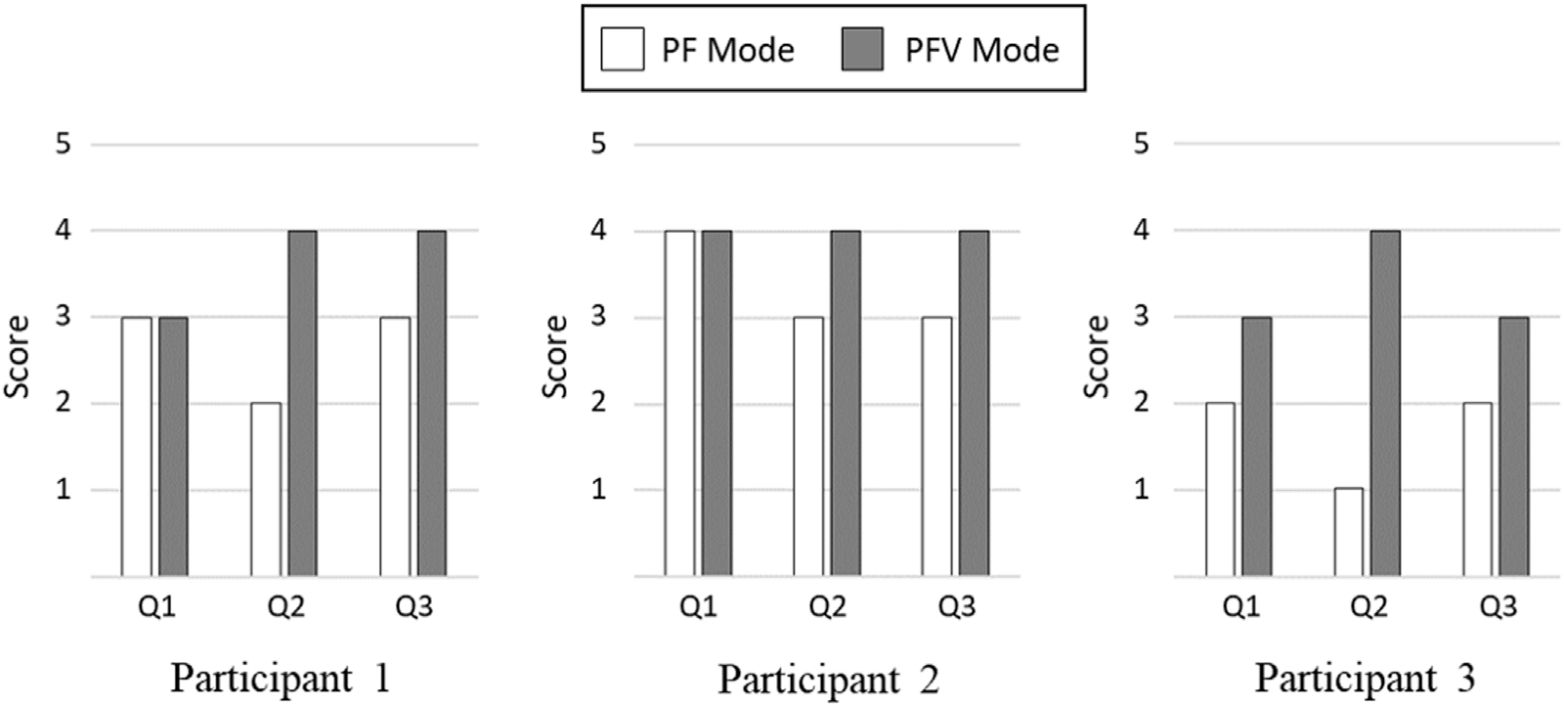
Comparison of Likert Scale Responses. (Q1) The gait motion felt natural. (Q2) The electrical stimulation of FES felt comfortable without causing much pain. (Q3) The gait experiment did not cause much muscle fatigue.

Figure 15 compares the Likert responses of the participants between the PF and PFV modes. The figure shows that there is no difference between the PF and PFV modes in terms of naturalness of gait motion (Q1), with the exception of Participant 3. In terms of comfortableness (Q2) and ease of muscle fatigue (Q3), however, the score with the PFV mode is higher than that with the PF mode for all the participants.

## 5 Concluding remarks

To validate the effectiveness of the developed system, we compared the joint angles and power generation at the ankle joint in two modes: PF mode (PAFO and FES without considering the volitional muscle activity of the patient) and PFV mode (PAFO and FES considering the volitional muscle activity of the patient). Figure 11 and Figure 12 demonstrate the effectiveness of the PFV mode in generating the ankle joint motion. Compared with the PF mode, the PFV mode is capable of generating a more powerful thrust at toe-off and ensures better foot-to-ground clearance during the swing phase. With the PFV mode, the range of motion (ROM) in plantar flexion is improved to allow post-stroke hemiplegic patients to stably control the walking speed by providing sufficient forward propulsion at the final stage of stance phase. Also, the PFV mode provides higher dorsiflexion during swing phase to help avoid foot drop that is very common after stroke.

Figure 13 and Figure 14 show the advantages of the PFV mode in generating torque and power at the ankle joint. The results demonstrate that, in the PFV mode, the power generated by the PAFO and FES can be significantly reduced while maintaining a sufficient level of total power generation. The experimental results show that in the PFV mode, the energy consumption by the PAFO and FES can be significantly reduced while adaptively assisting ankle motion depending on the volitional muscle activities of the patient. The developed system has the potential to improve the outcome of gait rehabilitation by inducing active patient participation depending on the stage of rehabilitation.

The results of subjective evaluation between the PF and PFV modes are demonstrated in Figure 15. While there is no significant difference in terms of naturalness of gait motion, the PFV mode received better rating than the PF mode in terms of comfortableness and ease of muscle fatigue.

In this study, we developed a novel hybrid system of PAFO and FES and proposed a coordination control method for the rehabilitation of patients with chronic hemiplegia. The PAFO assists the ankle joint motion based on the reference joint angle and impedance obtained from the biomechanical simulation. Unlike previous studies on motion optimization based on simulations of dynamic models of an exoskeleton robot and FES (Sharma et al., 2013; Chen et al., 2014), this study proposes the reference data obtained from the kinematic and dynamic simulations of the rectified biomechanical model. In this study, EMG data from healthy human participants was used to produce a natural simulation profile of FES. Based on the gait phase-based FSM algorithms (Quintero et al., 2010; Chang et al., 2017), continuous FES profiles can be provided for every step of the gait cycle. Also, the amplitude and duration of the FES profile are adaptively adjusted depending on the gait speed ranging from 1 to 5 km/h. A CNN-based estimation model predicts the joint torque and volitional torque for the coordination of the contributions of the PAFO and FES.

This study demonstrated the feasibility of the assist-as-needed rehabilitation system. The experimental results show that the coordination algorithm reduces energy consumption while maintaining the assistive effects as in previous studies (Del-Ama et al., 2014; Ha et al., 2015; Karavas et al., 2015). The results of this study suggest the potential for patient-driven gait rehabilitation of the developed system by considering the volitional movement and avoiding inefficient motor and FES activation.

The developed system can be clinically applied depending on the recovery stages of the post-stroke patient. In case the patient can barely generate voluntary contractions (before the late subacute phase, within 3 months of rehabilitation), the ankle movement is generated only by PAFO and FES as in the PF mode. In case the patient can generate sufficient volitional joint torque above a certain threshold level (rehabilitation in late subacute and chronic phase), the voluntary activity of the patient is considered in controlling PAFO and FES as in the PFV mode. This feature can encourage active participation of the patient in the rehabilitation process, which is essential for stroke rehabilitation.

Although this study focused on the assistance of ankle motion using a hybrid rehabilitation system for treadmill gait, further research is required to assist multi-DOF gait motion under various gait conditions. We are currently developing a modified version of this hybrid system using a knee-ankle-foot orthosis. In this study, we computed the reference gait pattern by using biomechanical simulation-based kinematic data, and we believe that the performance of the hybrid system can be further improved by using synchronized kinematic and EMG data collected from a large number of human subjects. In future studies, we plan to investigate the long-term therapeutic effects of the developed system on patients.

## Data Availability

The raw data supporting the conclusions of this article will be made available by the authors, without undue reservation.

## Abbreviations

PAFO, powered ankle-foot orthosis; FES, functional electrical stimulation; EMG, electromyography; TA, tibialis anterior; DOF, degree of freedom; ROM, range of motion; FSRs, force-sensitive resistors; DNN, deep neural network; 1D CNN, 1-dimensional convolutional neural network; MLP, multilayer perceptron; ReLU, rectified linear unit; Tanh, hyperbolic tangent.
